# Editorial: Non-Coding RNAs in Gastrointestinal and Gynecological Cancers: New Insights Into the Mechanisms of Cancer Therapeutic Resistance

**DOI:** 10.3389/fcell.2022.893868

**Published:** 2022-05-13

**Authors:** Peixin Dong, Feng Wang, Lei Chang, Junming Yue

**Affiliations:** ^1^ Department of Obstetrics and Gynecology, Hokkaido University School of Medicine, Hokkaido University, Sapporo, Japan; ^2^ Department of Laboratory Medicine, Affiliated Hospital of Nantong University, Nantong, China; ^3^ State Key Laboratory of Radiation Medicine and Protection, School of Radiation Medicine and Protection, Collaborative Innovation Center of Radiation Medicine of Jiangsu Higher Education Institutions, Medical College of Soochow University, Suzhou, China; ^4^ Department of Pathology and Laboratory Medicine, University of Tennessee Health Science Center, Memphis, TN, United States; ^5^ Center for Cancer Research, University of Tennessee Health Science Center, Memphis, TN, United States

**Keywords:** non-coding RNA, microRNA, long non-coding RNA, circular RNA, chemoresistance, cancer stem cells, gastrointestinal cancer, gynecological cancer

## Background

Chemoresistance is mediated by a variety of molecular mechanisms, including inhibition of drug influx, increased drug efflux, evasion of cell apoptosis, enhanced repair of DNA damage, induction of epithelial-mesenchymal transition (EMT), activation of cancer stem-like features, induction of autophagy, alterations in cancer metabolism, mutations of drug targets, and dynamic interactions between tumor cells and their surrounding microenvironment (Holohan et al., 2013). Tumor cells can become resistant to chemotherapeutic agents through a combination of different mechanisms (Vasan et al., 2019). Non-coding RNAs (ncRNAs), such as microRNAs (miRNAs), long non-coding RNAs (lncRNAs), and circular RNAs (circRNAs), have been implicated in chemoresistance (Wang et al., 2019).

In this research topic, 21 papers (including thirteen reviews and eight original research articles) that have shed light on the therapeutic resistance mechanisms controlled by non-coding RNAs in human gastrointestinal and gynecological cancers were chosen. The latest findings presented in this Research Topic add to our understanding of the mechanisms by which non-coding RNAs govern cancer cell resistance to chemotherapy, and may also point to meaningful molecular targets that could be utilized to eliminate chemoresistance and increase the survival of cancer patients.

## MiRNA and Chemoresistance

Tumor cells frequently have a high rate of glucose metabolism, and aerobic glycolysis has been linked to chemoresistance in tumors (Marcucci and Rumio, 2021). Hexokinase 2 (HK2), an enzyme that catalyzes the first committed step of glucose metabolism, has additional non-enzymatic roles in contributing to tumor chemoresistance (Dong et al., 2021). Yang et al. now report that HK2 is highly expressed in cervical cancer tissues, and its overexpression is correlated with poor patient survival. The authors found that HK2-knockdown cells formed fewer spheres than control cells, and knocking down HK2 markedly increased the susceptibility of cervical cancer cells to cisplatin. Furthermore, downregulation of the tumor suppressor miR-148a is essential for HK2 overexpression. Upregulation of miR-148a impairs the sphere-forming ability of cervical cancer cells and reduces cisplatin resistance. Hence, the combination of cisplatin and miR-148a mimic may be an effective strategy for preventing cancer stem cell-like properties and overcoming cisplatin resistance in cervical cancer patients.

## LncRNA and Chemoresistance

ATP binding cassette transporter proteins are well known for their contributions to chemoresistance by removing anti-cancer drugs from cancer cells. ATP Binding Cassette Subfamily C Member 10 (ABCC10, also known as MRP-7) plays a major role in this process (Szakács et al., 2006). A study by Huang et al. revealed that MRP-7 is expressed at high levels in drug-resistant endometrial cancer cell lines, and MRP-7 overexpression confers paclitaxel resistance to endometrial cancer cells while also promoting cell invasiveness *in vitro*. The authors further discovered that miR-98 is a key upstream suppressor of MRP-7 and that miR-98 directly targets *MRP-7* mRNA to repress MRP-7 expression. As a result, forced expression of miR-98 significantly reverses paclitaxel resistance and reduces the invasion of endometrial cancer cells. The authors found that lncRNA NEAT1 elevates MRP-7 levels by decreasing miR-98 expression. Thus, NEAT1/miR-98/MRP-7 signaling may be exploited as a critical therapeutic target to overcome paclitaxel resistance in endometrial cancer.

In endometrial cancer, DCST1-AS1 was discovered as an oncogenic lncRNA that promotes cancer progression by regulating the miR-665/HOXB5 pathway and the miR-873-5p/CADM1 pathway (Wang et al.).

Using an RNA binding protein immunoprecipitation assay, Trujano-Camacho et al. report that lncRNA HOTAIR interacts with β-catenin in the cervical cancer cell line HeLa. Their results demonstrate that the interaction between HOTAIR and β-catenin might hinder ICRT14 (a novel potent inhibitor of the Wnt/β-catenin pathway) from having anti-tumor effects on cell viability. The authors conclude that HOTAIR knockdown synergizes with ICRT14 to cause necrotic death in HeLa cells.

Qian et al. describe that lncRNA MALT1 is highly expressed in colorectal cancer tissues and cell lines, and MALT1 induces the proliferation and migration of colorectal cancer cells. Mechanistically, MALT1 promotes colorectal cancer progression via the miR-375/miR-365a-3p/NF-κB axis. They propose that targeting MALT1 might be an attractive treatment option for colorectal cancer.

## CircRNA and Cancer Progression

Li et al. report that circDNMT1, a circular RNA overexpressed in gastric cancer, can sponge miR-576-3p and subsequently elevate the levels of hypoxia-inducible factor-1 alpha (HIF-1α) in gastric cancer cells. By regulating the miR-576-3p/HIF-1α axis, circDNMT1 promotes gastric cancer cell proliferation, migration, invasion, and glycolysis.

## Computational Studies of ncRNAs

An original article by Wang et al. highlighted the importance of an immune-related lncRNA signature in predicting the prognosis of patients with colon cancer. Ding et al. confirmed that the expression of RP4-616B8.5, RP11-389G6.3, and CTD-2377D24.6 was increased in endometrial cancer tissues and that this 3-lncRNA signature was closely associated with histological subtype and advanced clinical stage in endometrial cancer patients. Their results indicate that this new lncRNA model might be useful for predicting the prognosis of patients with endometrial cancer.

## Reviews

13 review articles focus on the recent advances in the ncRNA research field and summarize the function and mechanisms of miRNAs, circRNAs, and lncRNAs in the pathogenesis of gastrointestinal and gynecological cancers. Zhao et al. outline the important impacts of miR-30d-5p on many types of malignancies, indicating that miR-30d-5p has broad potential as a diagnostic and therapeutic target for tumors. Ma et al. discuss the potential use of circRNAs as biomarkers or therapeutic agents for gynecological cancers. Wang et al. summarize the mechanisms underlying the involvement of circRNAs in modulating gastrointestinal cancer chemoresistance. Furthermore, Ghafouri-Fard et al. summarize the tumor suppressor role for a circRNA circITCH in various cancers. All these studies improve our understanding of the underlying mechanisms of ncRNAs and may aid in the development of new therapies for gastrointestinal and gynecological cancers.

## Summary

Taken together, the discovery of novel non-coding RNAs involved in the regulation of glycolysis or drug efflux has revealed fresh insights into the molecular basis of chemoresistance in gynecological cancers. Aberrant regulation of the miRNA-mRNA pathway, or the lncRNA/circRNA-miRNA-mRNA ceRNA network, contributes to chemoresistance in gastrointestinal and gynecological cancer cells. Notably, lncRNAs can interact with RNA-binding proteins and some RNA-binding proteins have critical roles in circRNA biogenesis. Further research on the interplay between ncRNAs and their molecular partners might provide opportunities for more effective strategies to prevent or overcome chemoresistance in gastrointestinal and gynecological cancer patients.
